# Pulmonary fibrosis complicated by lung cancer: bibliometric analysis from 2004 to 2024 - research status, trends and future directions

**DOI:** 10.3389/fimmu.2025.1514831

**Published:** 2025-04-03

**Authors:** Boyang Li, Fan Wu, Xinlai Ma, Weishan Yuan, Jiaqing Li, Wei Zhang, Xue Liu

**Affiliations:** ^1^ The First Clinical Medical College, Shandong University of Traditional Chinese Medicine, Jinan, China; ^2^ Institute of Traditional Chinese Medicine, Shandong University of Traditional Chinese Medicine, Jinan, China; ^3^ Department of Pulmonary and Critical Care Medicine, Affiliated Hospital of Shandong University of Traditional Chinese Medicine, Jinan, Shandong, China

**Keywords:** pulmonary fibrosis, nintedanib, lung cancer, pirfenidone, immunotherapy, bibliometrics

## Abstract

**Objective:**

Although research on the association between pulmonary fibrosis and lung cancer is of great significance, to date, no bibliometric analysis has been conducted on the comorbidity of these two diseases. This study aims to explore the current status and cutting - edge trends in this field through bibliometric analysis, and to establish new directions for future research.

**Methods:**

Using the Web of Science Core Collection database, statistical calculations, graphic, and data visualization tools such as CiteSpace, VOSviewer, and Biblimatrix - biblioshiny were adopted.

**Results:**

A total of 2,234 original Articles and Reviews on pulmonary fibrosis complicated by lung cancer published between 2004 and 2024 were identified. A slow growth trend in publications related to pulmonary fibrosis complicated by lung cancer was observed. The United States, Japan, and China were the countries with the greatest contributions. Professor Michael Kreuter from Marienhaus Clinic, Mainz, Germany, and the University of Michigan published the most articles. Through cluster analysis of co - cited literature, five main clusters were identified. Keyword analysis predicted that “nintedanib”, “pirfenidone”, “immunotherapy”, etc. might become hot topics in the field of the comorbidity of pulmonary fibrosis and lung cancer.

**Conclusion:**

This bibliometric analysis shows that the literature related to the comorbidity of pulmonary fibrosis and lung cancer is on a continuous upward trend. The research hotspots and trends identified in this study provide a reference for in - depth research in this field, aiming to promote the development of research on the comorbidity of pulmonary fibrosis and lung cancer.

## 1 Introduction

Pulmonary fibrosis and lung cancer are two significant pulmonary diseases with high global morbidity and mortality rates, posing a considerable threat to people’s lives and health. In recent years, the incidence of lung cancer has been on the rise year by year. The latest GLOBOCAN data from the International Agency for Research on Cancer shows that in 2020, the global incidence rate of all malignant tumors was 11.4%, and the mortality rate was 18%. It is estimated that there were 2.2 million new lung cancer cases worldwide (1–4). Moreover, some scholars predict that the age - standardized incidence rates of lung cancer will increase with the years in the next few years (5). Pulmonary fibrosis is an infectious or traumatic injury caused by various lung injuries, including oxidative stress, autoimmunity, vascular remodeling, drugs, etc. (6). Besides the impact on health, both pulmonary fibrosis and lung cancer can impose substantial economic costs on patients, bringing a heavy economic burden to patients, their families, and even society as a whole. Therefore, promoting research on pulmonary fibrosis, lung cancer, and their comorbidities is of great significance for safeguarding life and property health and reducing the economic burden on families and society. In recent years, with the development of research on pulmonary fibrosis and lung cancer, many aspects are facing opportunities and challenges. The research on the comorbidity of the two is facing increasingly complex problems. Although the information collected from bibliometrics can also provide a basis for formulating clinical guidelines (7, 8), there is currently no bibliometric study on the comorbidity of pulmonary fibrosis and lung cancer, and there is a lack of measurement and statistical methods. Therefore, bibliometric analysis has become a potential and indispensable statistical approach.

Bibliometrics is a discipline that uses mathematical and statistical methods to quantitatively analyze information (9). It quantitatively analyzes the distribution structure and change rules of a certain field based on the published information. It has been reported that bibliometrics is used for trend analysis of subjects, research frontier analysis, and prediction of research hotspots (10, 11). It can also be used to evaluate the contributions and cooperative relationships of countries, institutions, journals, and authors to specific research topics.

In this study, the bibliometric analysis method was adopted to quantitatively analyze the literature on pulmonary fibrosis complicated by lung cancer, evaluate the research contributions and cooperation of different countries, institutions, and authors in the field of pulmonary fibrosis complicated by lung cancer, and visually demonstrate the importance of countries, institutions, authors, and keywords in the network visualization map. This study aims to reveal the research status and trends of pulmonary fibrosis complicated by lung cancer, predict future research hotspots, summarize the current academic authorities and achievements, identify hotspots and future directions, and provide a theoretical basis for the pathogenesis and clinical research of pulmonary fibrosis complicated by lung cancer.

## 2 Methodology

### 2.1 Data sources

**Figure 1:**
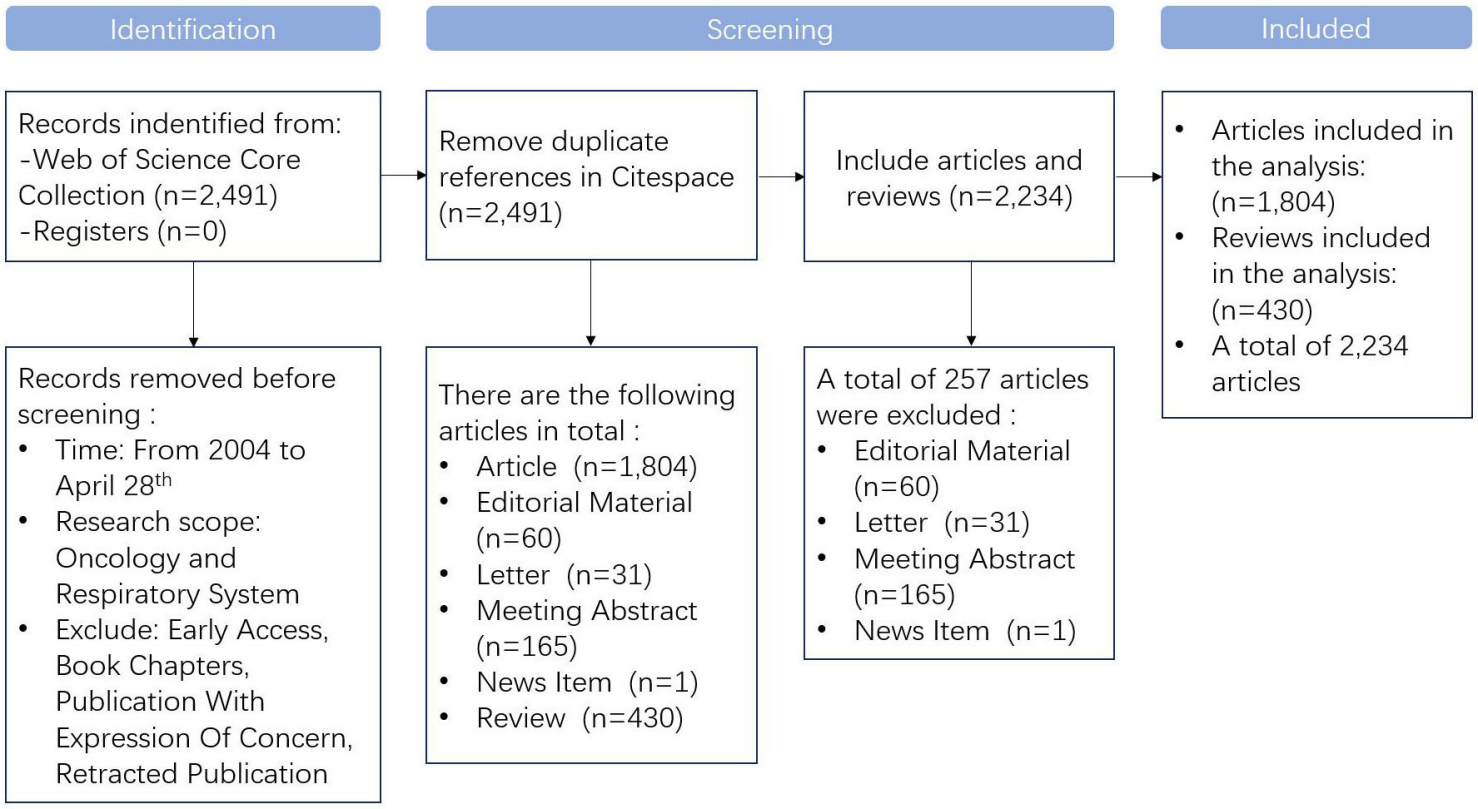
Literature screening process.

The Web of Science Core Collection (WOSCC), provided by Clarivate Analytics, is a globally renowned academic literature search and citation indexing database. It encompasses high - quality academic journals, conference papers, and other scholarly documents from diverse disciplinary fields worldwide. In this study, we carried out systematic literature retrieval and data extraction by searching for articles published between 2004 and 2024 in the Web of Science Core Collection (WoSCC). The search strategy employed was: TS=((pulmonary fibrosis) or (lung fibrosis) or (induced pulmonary fibrosis) or (bleomycin induced pulmonary fibrosis) or (rat pulmonary fibrosis) or (fibrosis) or (idiopathic pulmonary fibrosis) or (pulmonary interstitial fibrosis) or (mouse pulmonary fibrosis) or (bleomycin-induced pulmonary fibrosis in mice)) AND TS=((Lung Neoplasms) or (Pulmonary Neoplasms) or (Neoplasms, Lung) or (Lung Neoplasm) or (Neoplasm, Lung) or (Neoplasms, Pulmonary) or (Neoplasm, Pulmonary) or (Pulmonary Neoplasm) or (Lung Cancer) or (Cancer, Lung) or (Cancers, Lung) or (Lung Cancers) or (Pulmonary Cancer) or (Cancer, Pulmonary) or (Cancers, Pulmonary) or (Pulmonary Cancers) or (Cancer of the Lung) or (Cancer of Lung)), aiming to retrieve articles and reviews related to “pulmonary fibrosis and lung cancer”. As of April 28, 2024, all publications had been retrieved. Only English - language Articles and Reviews were included in the analysis. Other types of publications, such as reprints, book chapters, conference abstracts, and news items, were excluded (as depicted in **Figure 1**). We utilized CiteSpace to identify and eliminate duplicate publications, and then collected titles, abstracts, keywords, “countries”, “institutions”, “authors”, “journals”, and “references”. In total, 1,804 Articles and 430 Reviews were collected and analyzed.

### 2.2 Mapping tools

In this study, all 2,234 documents were analyzed using R (version 4.3.3), the R - bibliometrix package, Biblioshiny, Microsoft Office Excel 2016, VOSviewer (v.1.6.16), and CiteSpace (v.5.7.R5).VOSviewer is a software tool for constructing and visualizing bibliometric networks (12). It has powerful graphic capabilities and is suitable for handling large - scale data (13). CiteSpace was developed by Professor Chaomei Chen from Drexel University in the United States and is used for bibliometric analysis and data visualization (14, 15).We imported data from the WoSCC database. Biblioshiny, a website that provides a web interface for the R - bibliometrix package (16), was used for visual analysis of institutional publications, literature citations, and topic evolution. VOSviewer was employed to display the coupling relationships among authors, journals, and countries. CiteSpace was utilized to intuitively represent the basic knowledge and hotspots of pulmonary fibrosis and lung cancer, and to predict their research frontiers (17).

### 2.3 Map interpretation

In the mapping network, each node (Node, N) represents an object of analysis. A larger node graphic and a larger font for the node label both indicate a higher frequency of occurrence of the analyzed object. The color of the node, from top to bottom, represents the time span from the present to the year of the field data, with the top being the most recent. The lines (line, E) between nodes represent the collaborative relationships between the analyzed objects. Different colors of the connecting lines represent different years of initial collaboration. The line density (density) represents the density of collaboration. The betweenness centrality can reflect the importance of the research content in this field. Nodes with a betweenness centrality > 0.1 play a key role in the structure. In the cluster mapping, “#” represents a cluster. If the modularity value (Modularity Q, Q) of the cluster > 0.3, it means the cluster structure is significant. The S - value, that is, the average silhouette value (Silhouette, S), generally, when S > 0.5, the clustering is considered reasonable, and when S > 0.7, the clustering is convincing. In the burst analysis mapping, the red lines represent that the nodes have a high research popularity during that period of years.

## 3 Results

### 3.1 Document analysis

**Figure 2:**
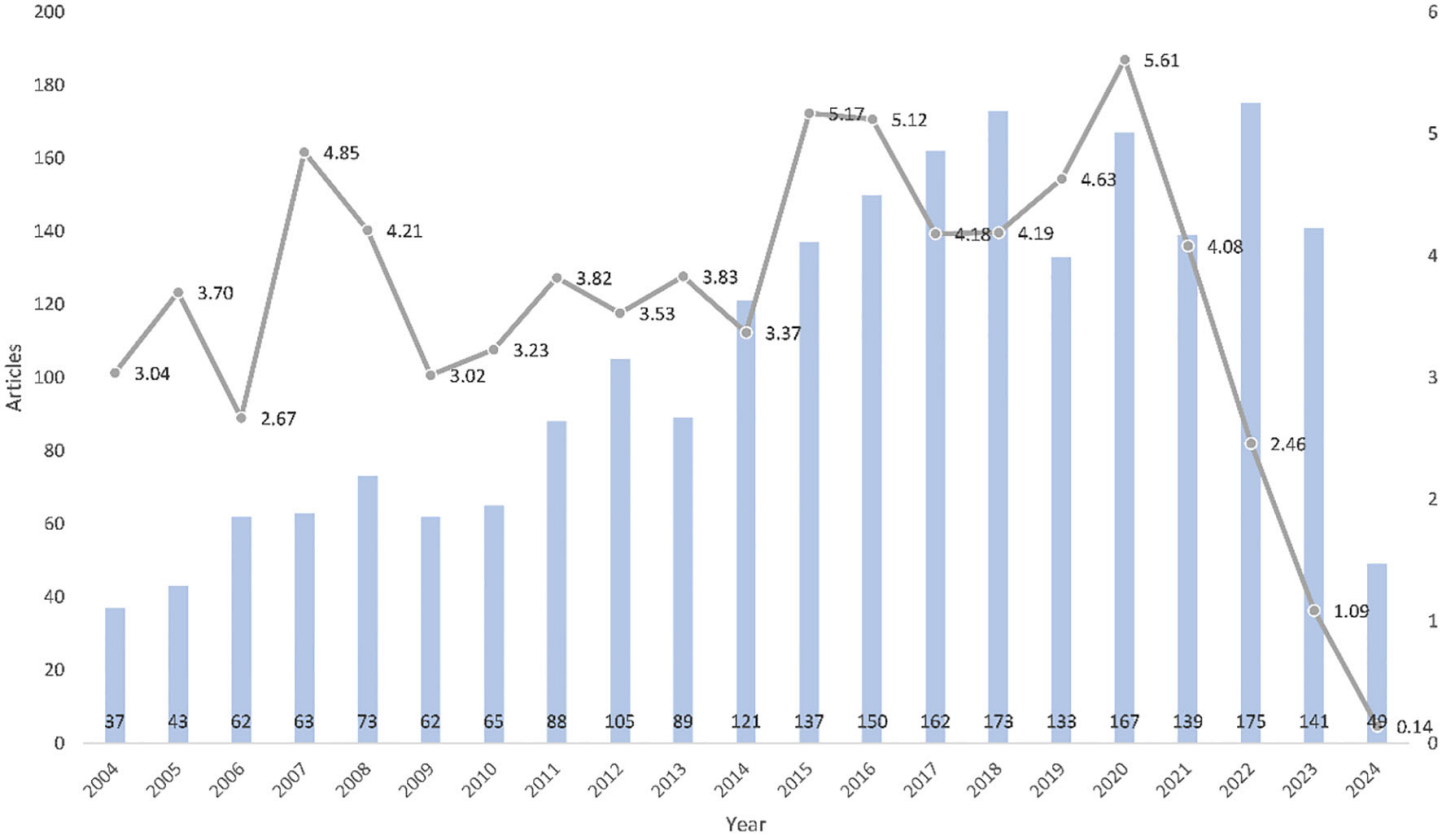
Chart of trends in the number of publications per year and the average year of citations.

The number of published papers (the blue bar chart in **Figure 2**) reflects, to a certain extent, the annual changes and future development trends of this research topic. The total average citations per year represent the average number of times each article related to the comorbidity of pulmonary fibrosis and lung cancer is cited annually (the gray line chart in **Figure 2**). This indicator reveals the overall academic impact of the articles published in the current year. In **Figure 2**, the horizontal axis represents the year, and the vertical axis represents the number of published papers. Regarding the number of published papers, from 2004 - 2010, the research on the comorbidity of pulmonary fibrosis and lung cancer in the academic community was in its infancy, with a relatively small number of papers published each year. Only 37 papers were published in 2004. Since 2010, the number of published papers has been on the rise year by year, reaching a peak in 2018 (173 papers), with an annual growth rate of 13.02%. In the following years, the number of published papers fluctuated slightly, but the range of fluctuations was not significant. This indicates that the comorbidity of pulmonary fibrosis and lung cancer has received continuous attention from the scientific community in recent years. However, as the data in this study only goes up to April 2024, we will not analyze the number of papers published in 2024.

It can be seen that the average number of citations reached its peak in 2020. The data in **Table 1** was obtained by using biblioshiny. In 2020, a total of 167 papers were published, with an average of 28.04 citations per paper. The average total citations per paper reached 5.61 times per year. Upon closer inspection, the peak in 2020 was attributed to the paper “Lung Cancer 2020: Epidemiology, Etiology, and Prevention” published by Brett C Bade in 2020 (18).

**Table 1:** Average citations per year (The “N” stands for the number of publications).

Year	Mean Title Citation per Article	Mean Title Citation per Year	N
2004	63.92	3.04	37
2005	73.91	3.70	43
2006	50.68	2.67	62
2007	87.35	4.85	63
2008	71.56	4.21	73
2009	48.31	3.02	62
2010	48.51	3.23	65
2011	53.41	3.82	88
2012	45.90	3.53	105
2013	45.99	3.83	89
2014	37.07	3.37	121
2015	51.68	5.17	137
2016	46.07	5.12	150
2017	33.41	4.18	162
2018	29.35	4.19	173
2019	27.80	4.63	133
2020	28.04	5.61	167
2021	16.30	4.08	139
2022	7.38	2.46	175
2023	2.19	1.09	141
2024	0.14	0.14	49

### 3.2 Country, institution, and author analysis

#### 3.2.1 Publishing countries

**Figure 3:**
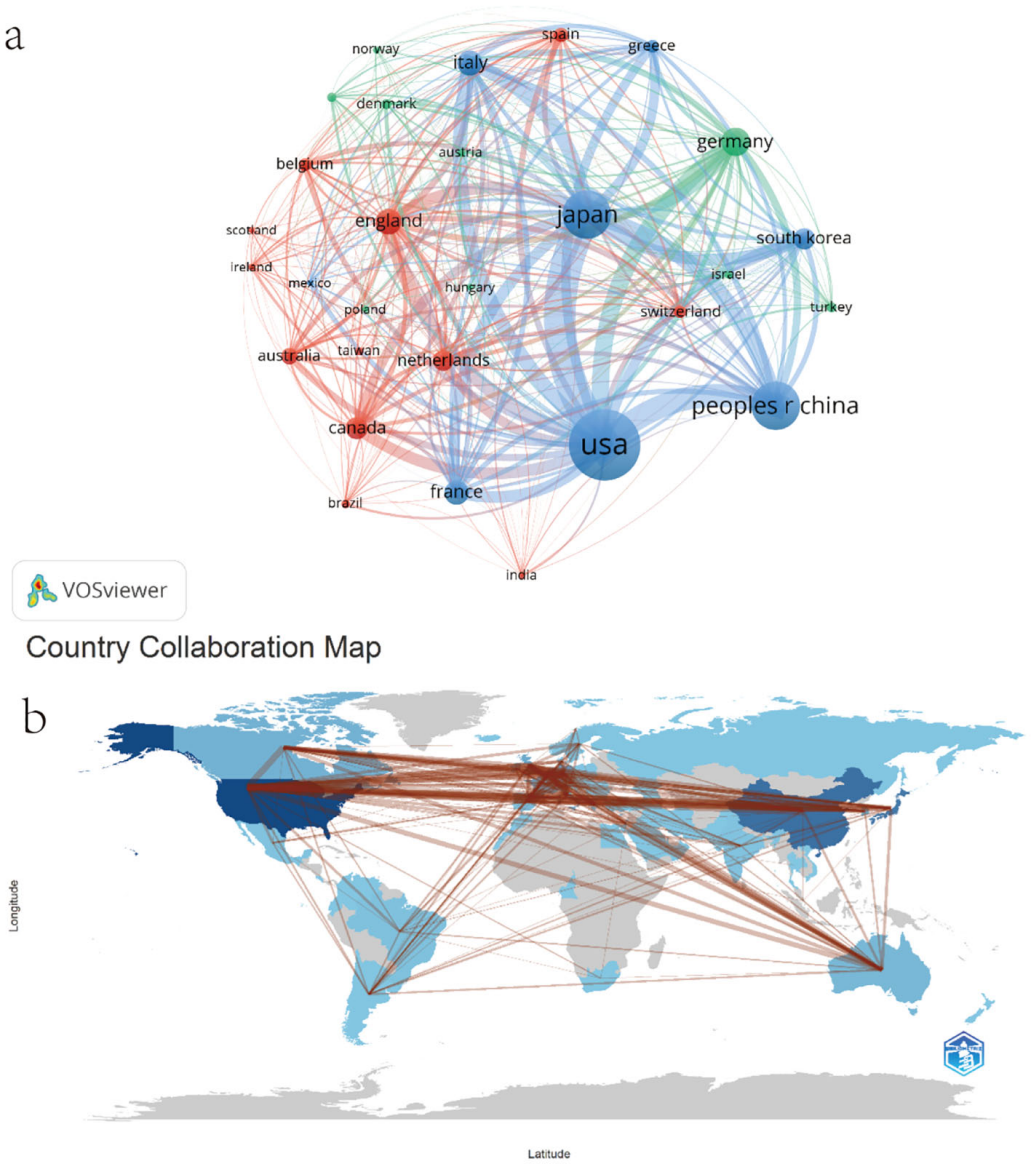
**(a)** National science achievement coupling network. **(b)** Country collaboration map.

Among all 79 countries and regions, we selected 29 countries with a minimum of 10 published papers for analysis. **Figure 3a** is a map of the number of papers published by each country based on document coupling, and **Figure 3b** is a map of cooperation among countries. **Table 2** shows the top ten countries in terms of the number of published papers. Regarding the scientific achievements of these countries (**Figure 3a**), the country with the largest number of published articles is the United States (the darkest blue in the figure), with 694 publications. The ten countries with the most research achievements (total number of publications listed in parentheses) are: the United States (694), Japan (382), the People’s Republic of China (382), Germany (164), England (138), Italy (135), France (129), Canada (108), South Korea (103), and the Netherlands (97). However, when analyzing the number of citations, the situation seems to change. Although the country with the largest number of citations is still the United States, with 37,297 citations (**Table 2**), the United Kingdom and Germany, countries with far fewer published papers than China, have a higher number of citations. Therefore, even though they rank slightly lower in terms of the number of publications, the large number of citations in the United Kingdom and Germany may demonstrate their importance in the research field.

**Table 2:** Top 10 countries in terms of number of publications.

**Rank**	**Country**	**Documents**	**Citations**	**Total link strength**
1	USA	694	37297	207047
2	JAPAN	382	11537	175339
3	PEOPLES R CHINA	382	8776	99537
4	GERMANY	164	8779	84718
5	ENGLAND	138	9465	82287
6	ITALY	135	5340	86611
7	FRANCE	129	4945	69827
8	CANADA	108	5101	62593
9	SOUTH KOREA	103	3759	55657
10	NETHERLANDS	97	5617	47910

#### 3.2.2 Publishing institutions and authors

**Figure 4:**
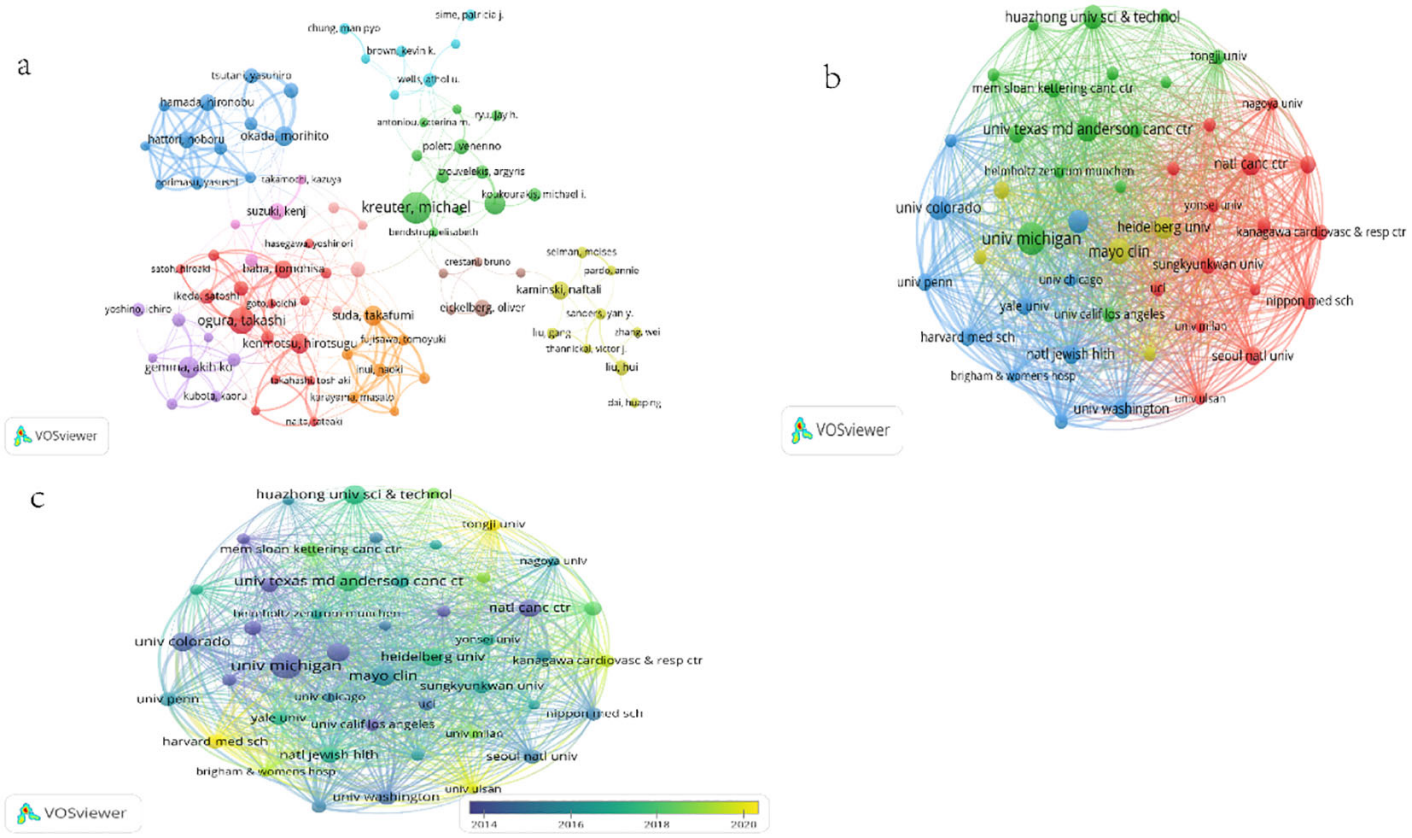
**(a)** Author collaboration Network. Each dot represents an author, and the dot size represents the number of articles published. The lines between the dots indicate collaboration between authors; The thickness of the line indicates the strength of the cooperation, and the thicker line indicates a stronger connection. **(b)** A collaborative network of major research institutions. The dots represent a research institution or group, while the size of the nodes represents the number of published papers. The curve between the nodes represents the connection between the two mechanisms; A thick line indicates a stronger connection between the two, as well as more collaborative articles. **(c)** A collaborative network superimposed by major research institutions and average time to publication. The colors in the graph from purple to yellow indicate the average publication time from far to near.

Over the past 20 years, a total of 13,759 authors have contributed to the research on the comorbidity of pulmonary fibrosis and lung cancer. **Figure 4** depicts the network of cooperative relationships among authors, and **Table 3** lists the top 10 authors who have published the most papers, Michael Kreuter from Marienhaus Clinic, Mainz, Germany, is the author who has published the most papers in the field of the comorbidity of pulmonary fibrosis and lung cancer, with 19 publications and 745 citations. Takashi Ogura from Kanagawa Cardiovascular Respiratory Center and Suresh Senan from University of Amsterdam Medical Center ranked second and third respectively, with 16 and 14 publications. However, when we analyzed based on the number of citations, the situation changed significantly (**Table 4**). Among the top 10 authors in terms of the number of publications, only Michael Kreuter (Rank: 4) and Suresh Senan (Rank: 2) ranked among the top 10 in terms of the number of citations among all authors. The author with the most citations is Oliver Eickelberg from the Department of Pulmonary and Critical Care Medicine. Co - authors play an important role in research innovation and result sharing. Researchers can improve their research through communication with other authors. Nevertheless, international and large - scale academic cooperation may be lacking due to language barriers and “isolation” issues.

**Table 3:** Top 10 most influential researchers in the field of pulmonary fibrosis and lung cancer comorbidities based on number of publications.

Author	Documents	Citations	Total link strength
kreuter, michael	19	745	15
ogura, takashi	16	361	59
senan, suresh	14	944	10
bouros, demosthenes	13	457	14
gemma, akihiko	13	442	34
kenmotsu, hirotsugu	12	406	47
suda, takafumi	12	395	52
okada, morihito	12	146	38
kaminski, naftali	11	472	6
baba, tomohisa	11	200	51

**Table 4:** The top 10 most influential researchers in the field of pulmonary fibrosis and lung cancer comorbidities based on citations.

Author	Documents	Citations	Total link strength
eickelberg, oliver	10	1120	4
senan, suresh	14	944	10
brown, kevin k.	7	878	4
kreuter, michael	19	745	15
thannickal, victor j.	6	740	12
palma, david a.	10	673	9
kudoh, shoji	8	646	18
sanders, yan y.	7	610	12
wells, athol u.	8	594	10
liu, gang	7	593	5

We used VOSviewer (**Figure 4b**) to include only institutions that had published at least 15 papers and subsequently analyzed 46 institutions. The cooperation among institutions is somewhat closer than that among countries. The top 12 institutions in terms of the number of papers are listed in **Table 5**. Ten of the most productive institutions are located in the United States, followed by one each in China and Germany. Notably, although the University of Washington has a relatively small total number of publications (23 papers), it leads in both citations and total link strength. This implies that the articles published by the University of Washington may be more persuasive. In terms of total link strength, National Jewish Health has the most links, with 11,377, indicating that it has the most collaborations with other institutions in the research on the comorbidity of pulmonary fibrosis and lung cancer. We used VOSviewer to color - code the institutions according to the average time required to generate an impact (**Figure 4c**); purple represents an earlier time, and yellow represents relatively recent years. Among them, emerging institutions such as Tongji University (Avg. pub. year: 2020.17), Harvard Medical School (Avg. pub. year: 2020.38), and University of Ulsan (Avg. pub. year: 2019.60) have also contributed commendable publications and collaborations in the field of the comorbidity of pulmonary fibrosis and lung cancer.

**Table 5:** The top 12 institutions in the number of publications.

organization	documents	citations	total link strength
University of Michigan	40	2775	11096
Mayo Clinic	31	1161	7528
UT MD Anderson Cancer Center	31	1396	3174
Huazhong University of Science and Technology	29	1241	3549
University of Colorado	29	1533	9602
University of Pittsburgh	28	2237	7759
Heidelberg University	27	865	9382
National Cancer Center	27	1098	5535
National Jewish Health	24	2088	11377
University of Washington	23	3159	10258
Memorial Sloan-Kettering Cancer Center	22	1190	4222
National Cancer Institute	22	1662	2843

### 3.3 Disciplines and journals analysis

**Figure 5:**
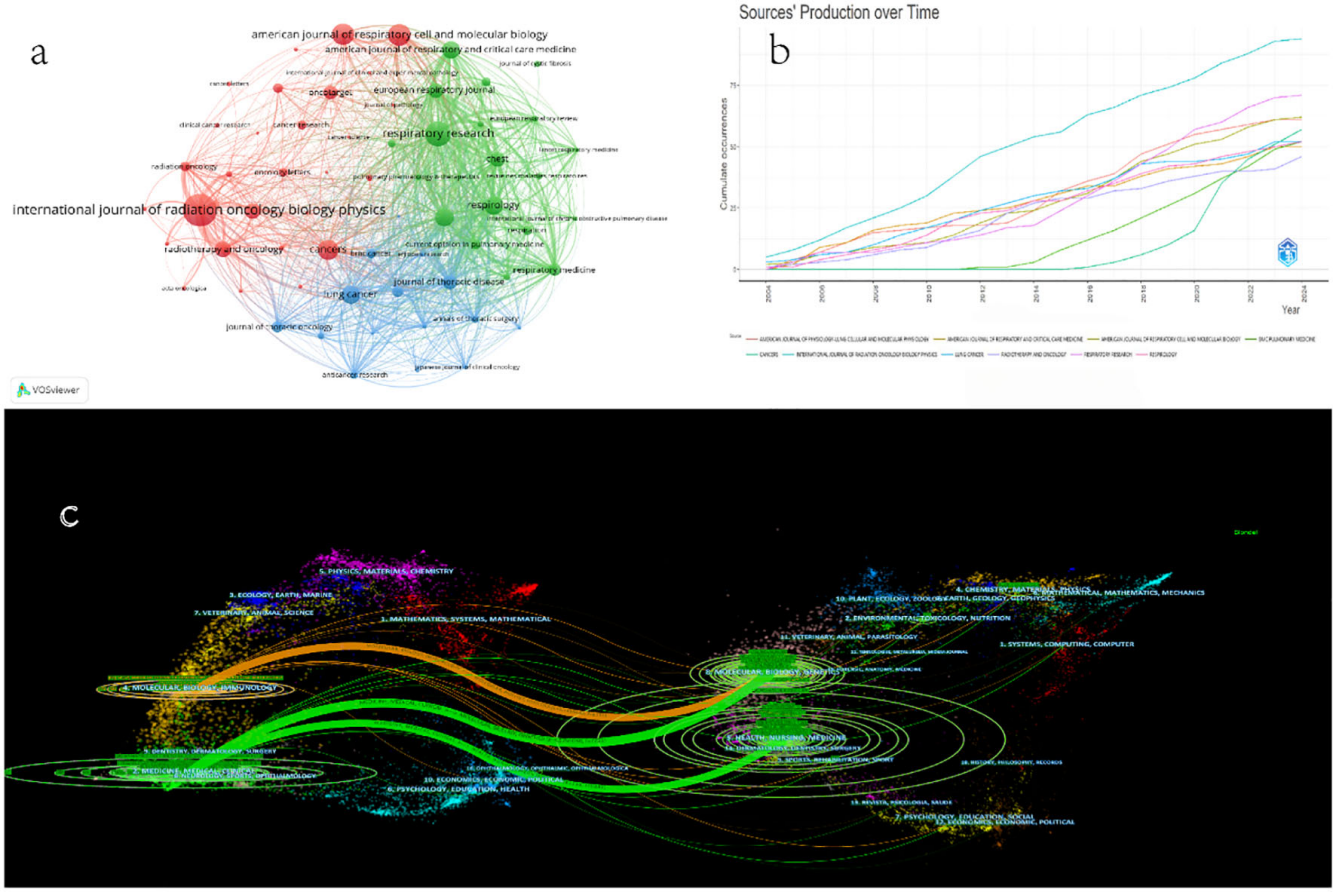
**(a)** Journal collaboration network based on bibliographic coupling. **(b)** A production map of the top 10 sources of publication over time in the field of pulmonary fibrosis and lung cancer comorbidities. **(c)** Double graph overlay of journals publishing literature on pulmonary fibrosis and lung cancer comorbidities. The ovals in the figure represent the number of publications corresponding to the journal. The longer the horizontal axis of the ellipse, the more authors it represents, and the longer the vertical axis of the ellipse, the more papers the journal has published.

**Figure 5a** lists the top 64 most common sources that publish articles in the field of the comorbidity of pulmonary fibrosis and lung cancer. The network construction is based on bibliographic coupling, and the relevance of items depends on the number of references they share. According to the number of literatures related to the comorbidity of pulmonary fibrosis and lung cancer published by each journal (**Table 6**), the top three are INTERNATIONAL JOURNAL OF RADIATION ONCOLOGY BIOLOGY PHYSICS, RESPIRATORY RESEARCH, and AMERICAN JOURNAL OF RESPIRATORY CELL AND MOLECULAR BIOLOGY. In **Figure 5a**, there are three journal clusters. The red cluster contains the most items (29 journals), including the most important journals. Additionally, the formation of smaller clusters can be identified, such as the green cluster (20 journals) and the blue cluster (15 journals). In **Figure 5b**, the number of annual publications is shown by journal. Each colored line represents a journal. INTERNATIONAL JOURNAL OF RADIATION ONCOLOGY BIOLOGY PHYSICS and RESPIRATORY RESEARCH are leading in the research on the comorbidity of pulmonary fibrosis and lung cancer. Although CANCERS started relatively late, it has been continuously publishing articles and eventually became a journal with significant influence in this field.

**Table 6:** Top 10 sources by volume of published literature.

Rank	Sources	Articles
1	INTERNATIONAL JOURNAL OF RADIATION ONCOLOGY BIOLOGY PHYSICS	94
2	RESPIRATORY RESEARCH	71
3	AMERICAN JOURNAL OF RESPIRATORY CELL AND MOLECULAR BIOLOGY	62
4	AMERICAN JOURNAL OF PHYSIOLOGY-LUNG CELLULAR AND MOLECULAR PHYSIOLOGY	61
5	CANCERS	57
6	BMC PULMONARY MEDICINE	52
7	LUNG CANCER	52
8	RESPIROLOGY	52
9	AMERICAN JOURNAL OF RESPIRATORY AND CRITICAL CARE MEDICINE	50
10	RADIOTHERAPY AND ONCOLOGY	46

The distribution of journals across various fields, the evolution of citation trajectories, and the shift of research centers can be illustrated using the double - map overlay of journals (19, 20). The result of the double - map overlay of journals (**Figure 5c** shows the position of the research on this topic relative to the major research disciplines. Each point on the map represents a journal, and the map is divided into two parts. The left - hand side is the citing map, and the right - hand side is the cited map. The curves are citation links, which fully demonstrate the ins and outs of citations. In the left - hand map, the circle represents the number of publications corresponding to a journal and shows the ratio of authors to the number of publications. The length of the ellipse represents the number of authors, and the width of the ellipse represents the number of publications (the more papers a journal publishes, the longer the vertical axis of the ellipse; the greater the number of authors, the longer the horizontal axis of the ellipse). The curves between the left and right parts of the map are citation links, and the trajectories of these links provide an understanding of the interdisciplinary relationships in this field. The z - Scores function highlights stronger and smoother trajectories, and higher scores are represented by thicker connecting lines. In this case, publications in fields such as Molecular, Biology, Genetics, Health, Nursing, Medicine are significantly influenced by publications in fields such as Molecular, Biology, Immunology, Medicine, Medical, Clinical.

In **Table 7**, we sorted these relationships in (**Figure 5c**) in descending order according to the z - scores. Each row is identified with the same color as the corresponding path shown in (**Figure 5c**). As described in **Table 7**, the most frequently cited areas are: (1) 2. Medicine, Medical, Clinical; (2) 4. Molecular, Biology, Genetics. These areas are mainly influenced by 8. Molecular, Biology, Genetics. At the top of the cited literature area in **Figure 5c**, publications in (1) 4. Chemistry, Materials, Physics, (2) 2. Environmental, Toxicology, Nutrition, and (3) 7. Psychology, Education, and Social also contribute to the citation pattern in this field to a certain extent, thus helping us understand the citation trends at the field level in the comorbidity of pulmonary fibrosis and lung cancer.

**Table 7:** Citation trends of papers in the field of pulmonary fibrosis and lung cancer comorbidities.

Color	Citing region	Cited region	Z-score
Green(above)	Medicine, Medical, Clinical	Molecular, Biology, Genetics	3.5237231
Yellow	Molecular, Biology, Immunology	Molecular, Biology, Genetics	2.716966
Green(below)	Medicine, Medical, Clinical	Health, Nursing, Medicine	2.6365754

### 3.4 Co-cited analysis

**Figure 6:**
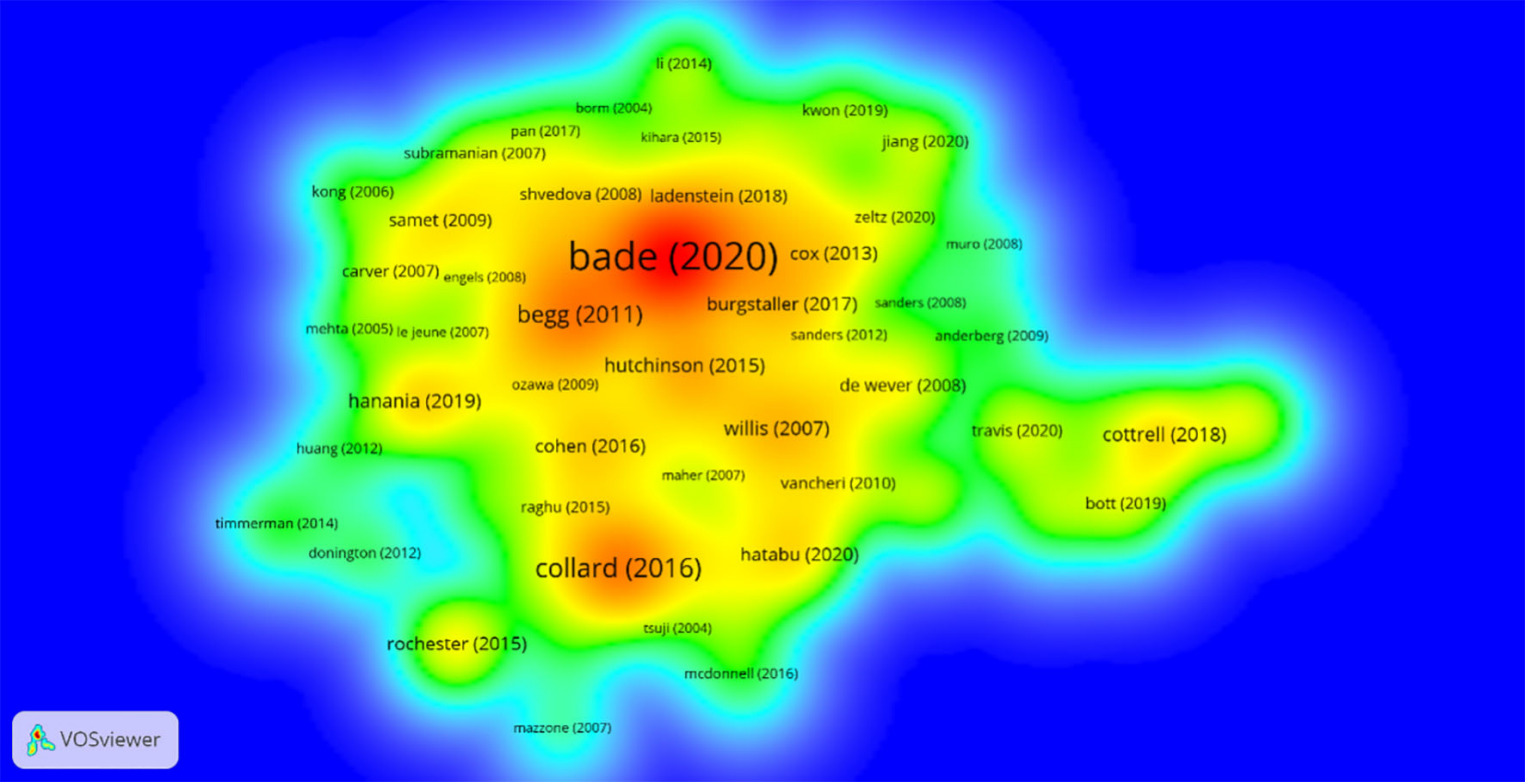
Density map of article co-cited analysis based on standardized citations.

One of the criteria for evaluating the quality of research papers is the number of times a paper is cited. Therefore, articles with a large number of citations are usually recognized by a large number of scholars, although this may not always be the case due to certain reasons. For the article citation analysis, the threshold was set at a minimum of 150 citations per article and having a large number of coupling relationships with other papers. Among the 2,234 articles surveyed, only 73 met this criterion. In addition, to correct the impact of the publication year of older literature, which gives some works an advantage in terms of citations over later works, we decided to generate a density map using “normal citations”. This algorithm divides the number of citations of a literature by the average of all articles published in the same year. **Figure 6** is the density map of the co - citation analysis of articles that meet the above conditions. It can be clearly seen from **Figure 6** that, based on normal citations, the most influential researchers are Bade, Begg, and Collard. These authors have made significant contributions to the research on the comorbidity of pulmonary fibrosis and lung cancer.

**Figure 7:**
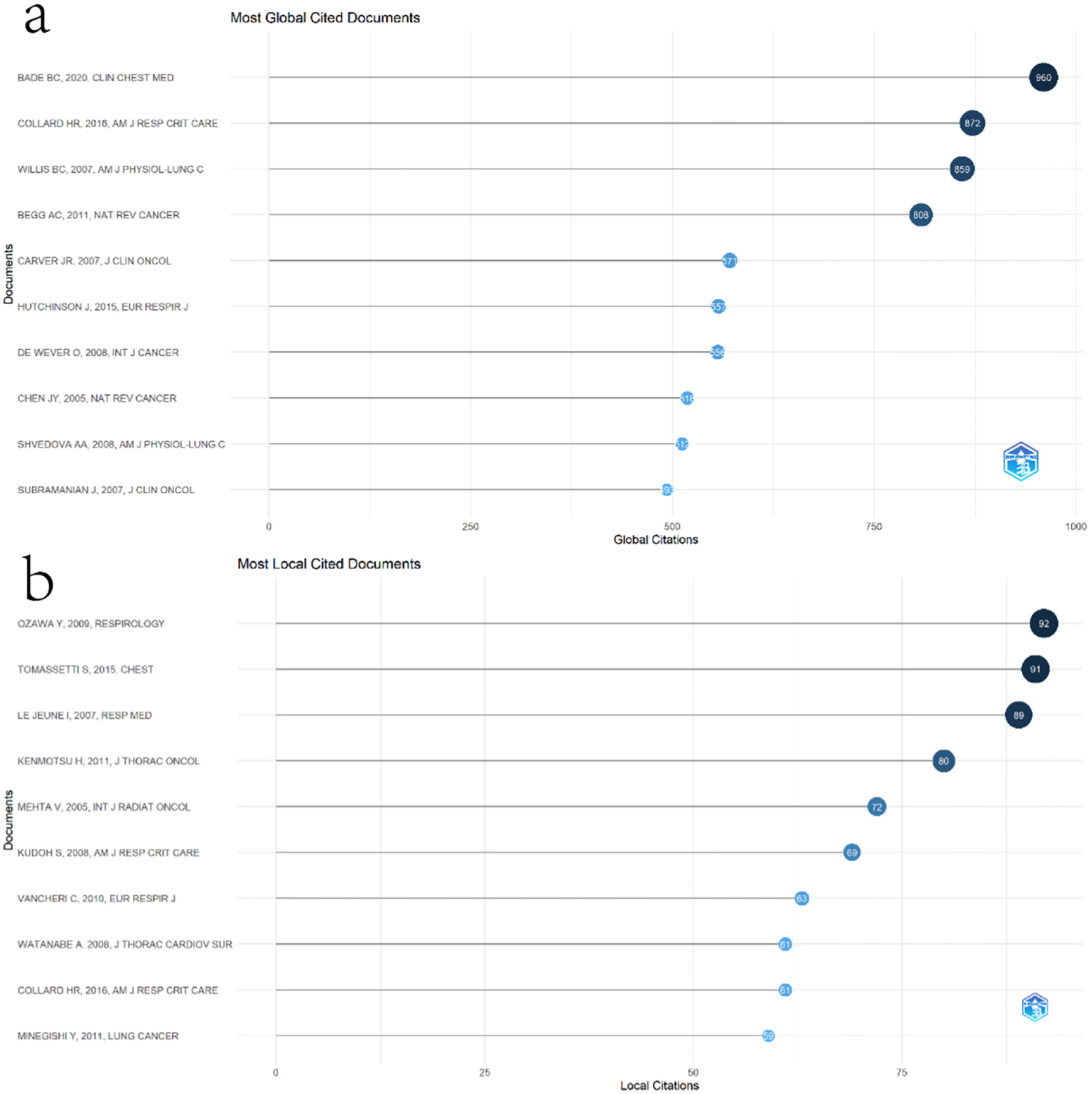
**(a)** Most global cited documents. **(b)** Most local cited documents.

We then ranked the included papers based on the number of citations in the WoSCC citation data (**Figure 7a**). The paper with the most citations was written by Brett C Bade from Yale University School of Medicine (960 citations). We also evaluated the impact of each paper in this field by calculating the number of local citations in the current dataset (**Figure 7b**). The ranking of local cited documents is slightly different from the total citation ranking. The paper ranked first in local cited documents is “Cumulative incidence of and predictive factors for lung cancer in IPF” written by Yuichi OZAWA.

### 3.5 Research hotspots

#### 3.5.1 Keyword analysis

**Figure 8:**
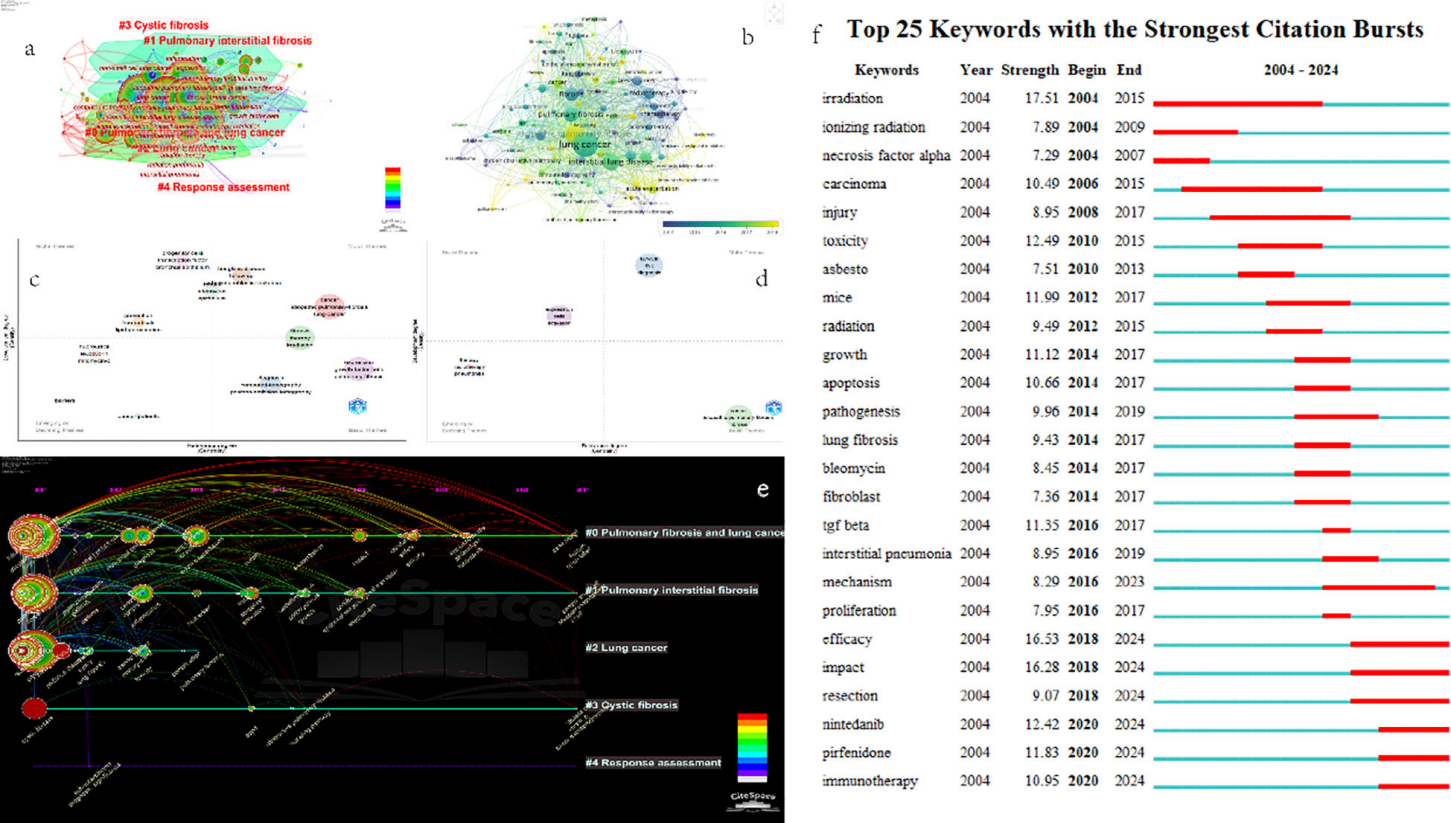
**(a)** 2004-2024 keyword clustering network graph. **(b)** Map of average year of occurrence of keywords. **(c)** Author keyword Thematic map from 2004 to 2010. **(d)** Author keyword Thematic map from 2010 to 2024. **(e)** The timeline visualization map in Citespace describes clusters distributed along a horizontal timeline, with each cluster shown from left to right. A legend of the release time is displayed at the top of the view. Cluster - The timeline is arranged vertically, in descending order of its size. The largest cluster is displayed at the top of the view. **(f)** Outbreak keyword map analysis.

The keyword clustering in **Figure 8a** was analyzed by CiteSpace software. The time slice was set from January 2004 to April 2024, with a span of 2 years per slice. The selection criteria was “Top 40”, the node type was “keyword”, and the calculation method was “LLR”. Other parameters were set as default values. The results are shown in **Figure 3** as follows: A total of 138 nodes and 1053 links are displayed, forming 5 clusters. The Q - value is 0.3507 > 0.3, indicating a significant clustering structure. Additionally, the S - value is 0.7801 > 0.7, making the clustering results convincing.

**Figure 8a** shows five main clusters: “ Pulmonary fibrosis and lung cancer “, “ Pulmonary interstitial fibrosis “, “ Lung cancer “, “ Cystic fibrosis “, and “Response assessment”. Here, the size refers to the capacity of the cluster. When the cluster capacity is less than 10, it indicates a poor clustering effect. Therefore, only the first three clusters were considered in this study. **Table 8** presents the top 10 keywords in terms of occurrence frequency, and **Table 9** shows the top 10 keywords in each cluster. The main keywords of #Cluster0 include “lung cancer”, “idiopathic pulmonary fibrosis”, “interstitial lung disease”, etc.; the main keywords of #Cluster1 include “fibrosis”, “pulmonary fibrosis”, “expression”, etc.; and the main keywords of #Cluster2 include “cancer”, “radiotherapy”, “therapy”, etc.

**Table 8:** Top 10 keywords based on number of occurrences.

Rank	keyword	occurrences	total link strength
1	lung cancer	376	495
2	idiopathic pulmonary fibrosis	237	305
3	interstitial lung disease	189	293
4	pulmonary fibrosis	142	157
5	fibrosis	119	130
6	radiotherapy	100	143
7	non-small cell lung cancer	97	128
8	radiation pneumonitis	73	101
9	breast cancer	70	65
10	acute exacerbation	67	151

**Table 9:** Top 10 keywords of each cluster.

Rank	Cluster0	Cluster1	Cluster2	Cluster3	Cluster4
1	lung cancer	fibrosis	cancer	cystic fibrosis	adenocarcinoma
2	idiopathic pulmonary fibrosis	pulmonary fibrosis	radiotherapy	copd	prognostic significance
3	interstitial lung disease	expression	therapy	signaling pathway	
4	disease	inflammation	pneumonitis	obstructive pulmonary disease	
5	survival	cell	breast cancer	tumor microenviron-ment	
6	risk	activation	cell lung cancer	stellate cell	
7	chemotherapy	lung	radiation therapy	invasion	
8	acute exacerbation	fibroblast	radiation pneumoniti		
9	diagnosis	tgf beta	gene expression		
10	mortality	mechanism	growth factor beta		

Thematic evolution analysis was proposed by Cobo et al. It can be used to detect, quantify, and visualize specific research fields, and visually display the thematic evolution in recent years. Similarly, taking 2010 as the demarcation point, this study was conducted using the bibliometrix software package. The Walktrap algorithm was applied to analyze the thematic evolution from 2004 to 2024, and then the Thematic maps shown in **Figures 8c**, **d** were generated. The strategic map is divided into four quadrants. The horizontal axis represents the central position of keywords, and the vertical axis represents density. Centrality indicates the degree of connection between clusters, and density represents the degree of connection among keywords within a cluster. Each circle represents a cluster, and the larger the area of the circle, the higher the frequency of keywords in the cluster. According to the definition of Cobo et al., the first quadrant contains keyword clusters that are well - developed, have strong centrality and high density, and are of great significance to the research on the comorbidity of pulmonary fibrosis and lung cancer. The second quadrant includes keyword clusters that are well - developed but not important for the current field. The third quadrant consists of less - developed keyword clusters. The themes in this quadrant have low density and low centrality, indicating that the keyword clusters in this quadrant are emerging or gradually disappearing. The themes in the fourth quadrant are important for the research field, but they are under - developed and are usually some basic keywords.

Subsequently, we used CiteSpace to analyze the timeline graph of keyword clusters (**Figure 8e**). In the graph, large nodes or nodes with red dendrograms are either highly cited, have burst citations, or both. One to three keywords with the highest frequency of occurrence in a specific year are shown below each timeline. By observing the graph, we can find that “#0 Pulmonary fibrosis and lung cancer “ has always been a research hotspot in the comorbidity of pulmonary fibrosis and lung cancer. Although “#1 Pulmonary interstitial fibrosis “ had no research between 2016 - 2023, articles with keywords in this cluster reappeared in 2024. This means that the research content contained in #cluster1 may soon become a research hotspot again.

Based on the keyword co - occurrence visualization in CiteSpace, we analyzed the mutated keywords and summarized the top 25 keywords with the strongest citation bursts in the comorbidity of pulmonary fibrosis and lung cancer. As shown in **Figure 8f**, the blue line represents the timeline, and the red segments on the blue timeline indicate the emergence of keywords. The red lines at different positions correspond to different start years, end years, and burst durations. Notably, “irradiation” has the highest strength (17.51), followed by “efficacy” (16.53), “impact” (16.28), “toxicity” (12.49), etc. According to the start time of occurrence, we found that “irradiation”, “ionizing radiation”, and “necrosis factor alpha” emerged earlier and were the early focuses of attention. “Immunotherapy”, “pirfenidone”, and “nintedanib” are the current research frontiers of the comorbidity of pulmonary fibrosis and lung cancer, and they are already in the outbreak period.

#### 3.5.2 Reference co-citation

The co - citation analysis of references lists the top 10 co - cited references related to the comorbidity of pulmonary fibrosis and lung cancer (**Table 10**). The article titled “An Official ATS/ERS/JRS/ALAT Statement: Idiopathic Pulmonary Fibrosis: Evidence - based Guidelines for Diagnosis and Management” by Ganesh Raghu has the highest number of co - citations, reaching 281 times. This article mainly summarizes the latest evidence and guidelines regarding the diagnostic criteria, imaging evaluation, pulmonary function tests, histological diagnosis, and treatment strategies of idiopathic pulmonary fibrosis (IPF) from aspects such as the definition of IPF, epidemiology, risk factors, diagnosis, and disease course monitoring. It holds great significance in the research of pulmonary fibrosis. The article ranked second in the number of co - citations is “Lung cancer and cryptogenic fibrosing alveolitis. A population - based cohort study” (21). This article found that gefitinib is more likely to cause acute interstitial lung disease (ILD) than other chemotherapeutic drugs in the treatment of non - small cell lung cancer, and it has been cited as many as 143 times. The article “A phase 3 trial of pirfenidone in patients with idiopathic pulmonary fibrosis” published in the NEW ENGLAND JOURNAL of MEDICINE studied a phase 3 clinical trial in IPF patients and found that pirfenidone can slow down the disease progression in patients with idiopathic pulmonary fibrosis, mainly reflected in delaying the decline of FVC% and the decline in the six - minute walk test.

**Table 10:** The top 10 references related to pulmonary fibrosis and lung cancer comorbidities were cited.

Rank	Citation	Reference title	Year	Author	Journal
1	281	An Official ATS/ERS/JRS/ALAT Statement: Idiopathic Pulmonary Fibrosis: Evidence-based Guidelines for Diagnosis and Management	2011	Ganesh Raghu	AMERICAN JOURNAL OF RESPIRATORY AND CRITICAL CARE MEDICINE
2	143	Lung cancer and cryptogenic fibrosing alveolitis. A population-based cohort study	2000	R Hubbard	AMERICAN JOURNAL OF RESPIRATORY AND CRITICAL CARE MEDICINE
3	124	Efficacy and Safety of Nintedanib in Idiopathic Pulmonary Fibrosis	2014	Luca Ric-heldi	the NEW ENGLAND JOURNAL of MEDICINE
4	107	Diagnosis of Idiopathic Pulmonary Fibrosis. An Official ATS/ERS/JRS/ALAT Clinical Practice Guideline	2018	Ganesh Raghu	AMERICAN JOURNAL OF RESPIRATORY AND CRITICAL CARE MEDICINE
5	92	Cumulative incidence of and predictive factors for lung cancer in IPF	2009	Yuichi Ozawa	RESPIROLOGY
6	91	The impact of lung cancer on survival of idiopathic pulmonary fibrosis	2015	Sara Tomassetti	CHEST
7	89	A phase 3 trial of pirfenidone in patients with idiopathic pulmonary fibrosis	2014	Talmad-ge E King Jr	the NEW ENGLAND JOURNAL of MEDICINE
8	89	The incidence of cancer in patients with idiopathic pulmonary fibrosis and sarcoidosis in the UK	2007	Ivan Le Jeune	RESPIRATORY MEDICINE
9	84	American Thoracic Society/European Respiratory Society International Multidisciplinary Consensus Classification of the Idiopathic Interstitial Pneumonias. This joint statement of the American Thoracic Society (ATS), and the European Respiratory Society (ERS) was adopted by the ATS board of directors, June 2001 and by the ERS Executive Committee, June 2001	2002	American Thoracic Society	AMERICAN JOURNAL OF RESPIRATORY AND CRITICAL CARE MEDICINE
10	84	Lung cancer in patients with idiopathic pulmonary fibrosis	2001	J Park	EUROPEAN RESPIRATORY SOCIETY

Among the top 10 references in terms of citation count, three are joint statements or clinical practice guidelines issued by multiple societies (22–24). This indicates that these statements possess sufficient authority and can offer valuable reference. When conducting subsequent research on the comorbidity of pulmonary fibrosis and lung cancer, paying attention to these statements can provide ideas and directions for our studies.

## 4 Discussion

### 4.1 General information

Pulmonary fibrosis (PF) is the end - stage change of a large category of pulmonary diseases characterized by fibroblast proliferation, massive extracellular matrix aggregation, accompanied by inflammatory damage and destruction of tissue structure. That is, after normal alveolar tissue is damaged, abnormal repair leads to structural abnormalities. Its pathological features include damage and abnormal proliferation of alveolar epithelial cells, deposition of extracellular matrix (ECM), and proliferation and activation of fibroblasts (25–27), resulting in destruction of lung structure and loss of respiratory function. The causes of the vast majority of PF are unknown, and it is called idiopathic interstitial pneumonia. Under the category of pulmonary fibrosis, it can be further divided into several branches such as idiopathic pulmonary fibrosis, connective - tissue - disease - related pulmonary fibrosis, and drug - induced pulmonary fibrosis. The pathogenesis of various types of pulmonary fibrosis is complex. Regarding idiopathic pulmonary fibrosis (IPF), there have been many research progresses in its pathogenesis. It has shifted from the initial “inflammatory reaction theory” to a focus on the “injury - repair theory”. Results have confirmed that vascular remodeling plays an important role in pulmonary fibrosis diseases including IPF (28).

Lung cancer is the leading cause of cancer - related deaths in both men and women worldwide. Approximately 85% of patients are associated with smoking (29). The geographical differences in the incidence and mortality rates of lung cancer patients in different countries or regions are mainly attributed to the historical patterns of smoking and the maturity of the tobacco epidemic (30). Therefore, currently, smoking remains the primary factor contributing to the development of lung cancer (18). The pathogenesis of lung cancer is also highly complex, being the result of a multi - step process of cellular gene damage under the combined action of multiple factors. **Table 11** compares the etiology of pulmonary fibrosis and lung cancer.

**Table 11:** Comparison of pulmonary fibrosis and lung cancer.

Type of disease	Pathogenic factors
Pulmonary fibrosis	Smoking, drugs, occupational or household, radiotherapy, high concentration oxygen therapy, viral infections, gastroesophageal reflux, aging, genetic factors, disease (connective tissue diseases, vasculitis, etc.) factors
Lung cancer	Smoking, occupational and environmental exposures, air pollution, genetics and genetic alterations, diet and nutrition, ionizing radiation, others

### 4.2 Research Hotspots

We have classified the extracted keywords into five clusters and will discuss the first three major clusters, through which we aim to analyze the research hotspots in the field of the comorbidity of pulmonary fibrosis and lung cancer.

#Cluster0 is mainly focused on clinical research. This cluster is primarily concerned with the diagnosis and prognosis of diseases related to the comorbidity of pulmonary fibrosis and lung cancer. Research within this cluster explores the diagnosis and treatment of these two pulmonary diseases and their comorbidity from a macroscopic perspective. It can be observed that studies leaning towards clinical evaluation, such as those related to “survival,” “risk,” and “mortality,” account for a significant proportion. Moreover, terms like “acute exacerbation” and “diagnosis” further expand and complement the clinical understanding of acute conditions associated with the comorbidity of pulmonary fibrosis and lung cancer. Just as the thematic evolution analysis indicates, the current motor themes form a cluster consisting of “survival,” “risk,” and “diagnosis.” Therefore, ensuring the survival of patients with the comorbidity of pulmonary fibrosis and lung cancer will be one of the research hotspots in this field.

#Cluster1 mainly focuses on specific research of pulmonary fibrosis, while #Cluster2 is centered around specific research of lung cancer. There is a significant overlap between #Cluster1 and #Cluster2, which is highly relevant to the research theme of this article. Many of the pathogenic mechanisms and treatment methods for pulmonary fibrosis and lung cancer can be mutually explained and applied. For example, inhibiting the TGF - β pathway can alleviate the induction of both pulmonary fibrosis and lung cancer. Therefore, we can discuss #Cluster1 and #Cluster2 together.

In the research fields of lung cancer and pulmonary fibrosis, a key trend is emerging: exploring reliable biomarkers by studying signaling pathways, gene expression, etc. Since both pulmonary fibrosis and lung cancer often have insidious symptoms in the early stages, most patients are already in the middle or late stages at the time of diagnosis (31). The identification of biomarkers can significantly improve the early detection of pulmonary fibrosis and lung cancer, creating conditions for early discovery and treatment of the diseases. These biomarkers not only contribute to early detection but also have important predictive value. They can provide references for clinicians, assist in formulating treatment plans, and predict patients’ treatment responses, thus enabling more precise treatment.

To achieve dual attention to identification and prognosis, multidisciplinary collaboration is required. The knowledge and techniques of molecular biology, bioinformatics, and clinical science need to be organically integrated. Only in this way can the biomarkers discovered in the laboratory be successfully translated into effective tools in clinical practice. Meanwhile, this will remain an ongoing research hotspot in the field of the comorbidity of pulmonary fibrosis and lung cancer in the future.

In addition, in recent years, there has been an increasing amount of research on the tumor microenvironment and the fibrotic microenvironment. For instance, Chong Zhang et al. (32) discovered that the fibrotic microenvironment may enhance the metastatic seeding of tumor cells in the lungs by chemo - attracting tumor cells and inhibiting their apoptosis through the activation of the FN1/SPP1 - ITGAV signaling pathway. Gang Wang et al. (33) found that the anti - fibrotic drug pirfenidone can prevent cancer progression by suppressing the tumor microenvironment. Takuya Ueda et al. (34) have demonstrated that the tumor microenvironment of patients with common interstitial pneumonia - associated lung adenocarcinoma acquires an immunosuppressive state, indicating a certain degree of connection between the tumor microenvironment and the comorbidity of pulmonary fibrosis and lung cancer.

Pulmonary fibrosis (PF) is a chronic, progressive, fibrotic lung disease that can lead to irreversible decline in patients’ lung function, progressive respiratory failure, and even death. The pathogenesis of PF has not been fully elucidated. Current research indicates that PF is caused by persistent alveolar epithelial cell damage and abnormal repair, proliferation of fibroblasts, and accumulation of extracellular matrix, resulting in disordered lung structure and fibrosis.

Lung cancer is a type of cancer that begins when abnormal cells in the lungs grow in an uncontrolled manner. The research by Don C et al. (25) has found that macrophages play an important role in interstitial fibrosis, usually driven by the transforming growth factor - β (TGF - β) pathway. Various growth factors including PDGF, VEGF, FGF and their receptors are highly expressed in patients with pulmonary fibrosis or lung cancer, which can promote disease progression (35–38). Wnt/β - catenin is closely related to the progression and development of cancer (39) and fibrosis (40). TGF - β1 is involved in regulating cell proliferation, differentiation, apoptosis, adhesion, and movement, and is a key cytokine that promotes excessive production of extracellular matrix (ECM) and inhibits its degradation (41, 42). Inhibiting the expression of TGF - β can delay the deterioration of lung cancer and pulmonary fibrosis (43, 44).

At present, preliminary progress has been made in the research of targeted therapeutic drugs for the above - mentioned pathways. Multiple researchers (35, 36) have found that nintedanib can inhibit the activities of PDGF, VEGF, and FGF, and suppress the TGF - β and Wnt/β - catenin pathways, thereby delaying or controlling the disease progression of pulmonary fibrosis and lung cancer. Pirfenidone can also reduce the deposition of extracellular matrix by decreasing the expression of growth factors such as PDGF, stimulate fibroblast mitosis, prevent fibroblast proliferation, inhibit collagen synthesis and promote its degradation (45), and reduce the production of TNF - α, thus inhibiting the inflammatory response, tissue damage and necrosis, and suppressing the subsequent tissue repair and fibrosis processes (46). That is, it controls the conditions of pulmonary fibrosis and lung cancer by inhibiting the expression of TGF - β, PDGF, etc. (47).

However, the mechanisms of action of new drugs are extremely complex, and more of their mechanisms and safety remain to be further studied. Therefore, these aspects are also the current research focuses.

The tracking of research hotspots and trends is carried out through keyword burst detection (**Figure 8f**). Based on the top 25 keywords with the highest citation burst rates, the early research focused on “irradiation”, “ionizing radiation”, and “necrosis factor alpha”. In the middle section of the timeline, the keywords were related to basic research, such as “toxicity”, “mice”, and “apoptosis”. The most recent research has centered on “efficacy”, “impact”, “resection”, “nintedanib”, “pirfenidone”, and “immunotherapy”. This indicates that the research hotspots in the comorbidity of pulmonary fibrosis and lung cancer seem to have shifted from radiotherapy, which causes significant harm to the body, to oral medications and immunotherapy with less harm to the body. There is a greater emphasis on the efficacy and impact after treatment, rather than simply killing tumor cells at all costs. It also means that the research hotspots are transitioning from basic research to clinical research.

**Figure 8b** is a map showing how keywords change over the years. Keywords that emerged later are closer to the color yellow. Judging from **Figure 8c**, the keywords in the first quadrant, such as “cancer, idiopathic pulmonary - fibrosis, lung cancer” (red cluster); “fibrosis, therapy, irradiation” (green cluster); and “hodgkins - disease, follow - up, acute lymphoblastic - leukemia” (brown cluster), are the ones that have been studied more. This implies that during the period from 2004 to 2010, these topics played a significant role in the research on the comorbidity of pulmonary fibrosis and lung cancer. However, as depicted in **Figure 8d**, with the continuous in - depth research, the above - mentioned three keyword clusters have been classified into the third and fourth quadrants. This indicates that the research on the comorbidity of pulmonary fibrosis and lung cancer is no longer confined to basic research on certain diseases, and treatment methods such as radiotherapy are about to fade out of the researchers’ focus. Instead, “survival risk diagnosis” (blue cluster), which pays more attention to humanistic care, has become an important part of the research on the comorbidity of pulmonary fibrosis and lung cancer.

Although not explicitly pointed out in the above figures, in recent years, the relationship between Coronavirus Disease 2019 (COVID - 19) - induced Novel Coronavirus Pneumonia (NCP) and the comorbidity of pulmonary fibrosis and lung cancer deserves our full attention. Eusebi Chiner - Vives et al. (48) found that COVID - 19 has a significant negative impact on patients with pre - existing pulmonary fibrosis or lung cancer. Shao - Lin Tao et al. (49) discovered that COVID - 19 can also induce the occurrence of pulmonary fibrosis and lung cancer by causing immunodeficiency. Yang Li et al. (50) used bioinformatics and systems biology methods to identify 10 drugs, such as resveratrol, dasatinib, and decitabine, with potential activity in treating NCP - related comorbidity of pulmonary fibrosis and lung cancer. Resveratrol has been extensively studied in recent years. Ramli I et al. (51) found that resveratrol has multiple molecular therapeutic targets, including angiotensin - converting enzyme (ACE), and these targets have a rather positive impact on alleviating respiratory diseases (52). Research indicates (53, 54) that ACE is also one of the key nodes connecting pulmonary fibrosis and lung cancer. Multiple studies (55–57) suggest that various anti - tumor drugs, including dasatinib, act as potential inhibitors for treating NCP - related comorbidity of pulmonary fibrosis and lung cancer through nodes like ACE. Certainly, the mechanisms of action and clinical application effects of these drugs in treating NCP - related comorbidity of pulmonary fibrosis and lung cancer require more supporting research. This will also be an ongoing research hotspot in the future study of NCP - related comorbidity of pulmonary fibrosis and lung cancer.

The reasons why COVID - 19 and NCP - related keywords are not reflected in our visual maps may be their relatively short emergence time (appearing only after 2020) and the insufficient number of related literatures (with only a short outbreak period of 2 - 3 years). However, considering that there are still patients with NCP - related comorbidity of pulmonary fibrosis and lung cancer emerging, we believe that it is very necessary to discuss the relationship between NCP and the comorbidity of pulmonary fibrosis and lung cancer.

Based on the results of the literature analysis, we can conclude that future research directions will focus on drug experiments centered around signaling pathways, growth factors, etc., to study the improvement of the clinical survival of patients with the comorbidity of pulmonary fibrosis and lung cancer. Additionally, efforts will be made to explore and evaluate new therapeutic drugs for this comorbidity, aiming to find treatment measures with good clinical efficacy and minimal harm to patients. Many anti - tumor drugs also show good efficacy in treating pulmonary fibrosis, and vice versa. The future treatment strategies for the comorbidity of pulmonary fibrosis and lung cancer may involve combinations of multiple drugs targeting different aspects of both conditions.

### 4.3 Limitations

To ensure a high-quality bibliometric analysis, all analyses in this study were conducted solely based on articles from the Web of Science Core Collection (WoSCC) database, which is one of the most prominent scientific publication databases for many research topics. However, as studies not published in non-SCI journals or other databases were excluded, a significant number of studies were not included in the analysis. Additionally, Biblioshiny, VOSviewer, and CiteSpace cannot fully replace systematic retrieval. Third, bibliometrics cannot evaluate the quality of individual studies, as citation counts are time-dependent. This means that earlier publications may receive more citations than recently published ones, primarily due to their publication date (58). These limitations may have a minor impact on the overall results, but we have employed appropriate algorithms to optimize the visualization outcomes. Therefore, these limitations are unlikely to alter the main trends proposed in this study. Overall, our research lays a foundation for understanding the research themes, hotspots, and development trends in the comorbidity of pulmonary fibrosis and lung cancer.

## Data Availability

The original contributions presented in the study are included in the article/supplementary material.
